# Stress cardiac magnetic resonance imaging effectively reclassifies risk in patients with known or suspected stable coronary artery disease

**DOI:** 10.1186/1532-429X-15-S1-P186

**Published:** 2013-01-30

**Authors:** Jiazuo H Feng, Ravi Shah, Bobby Heydari, Venkatesh L Murthy, Siddique Abbasi, Tomas G Neilan, Ron ABlankstein, Marcelo Di Carli, Michael Jerosch-Herold, Raymond Y Kwong

**Affiliations:** 1 Brigham and Women's Hospital, Boston, MA, USA

## Background

A recent large-scale clinical trial has indicated that an initial invasive strategy does not improve cardiac outcomes beyond optimized medical therapy in patients with stable coronary artery disease (CAD). Therefore, novel methods to stratify at-risk patients may refine therapeutic decisions and improve outcomes. We sought to test the hypothesis that stress cardiac magnetic resonance (CMR) imaging effectively prognosticates and reclassifies patient risk in a consecutive clinical cohort of patients with known or suspected CAD across a spectrum of guideline-determined risk categories.

**Figure 1:**
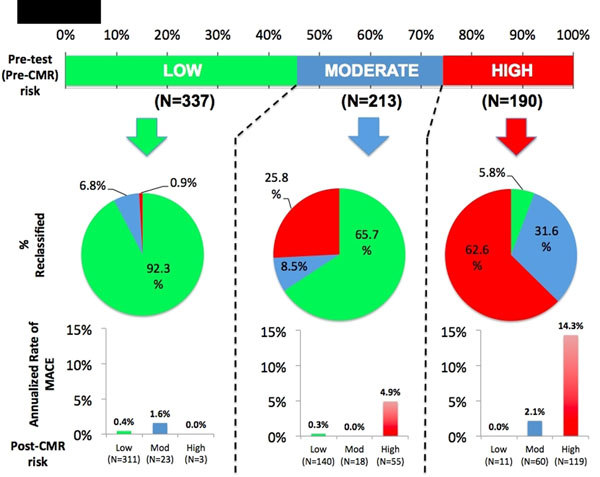


**Figure 2:**
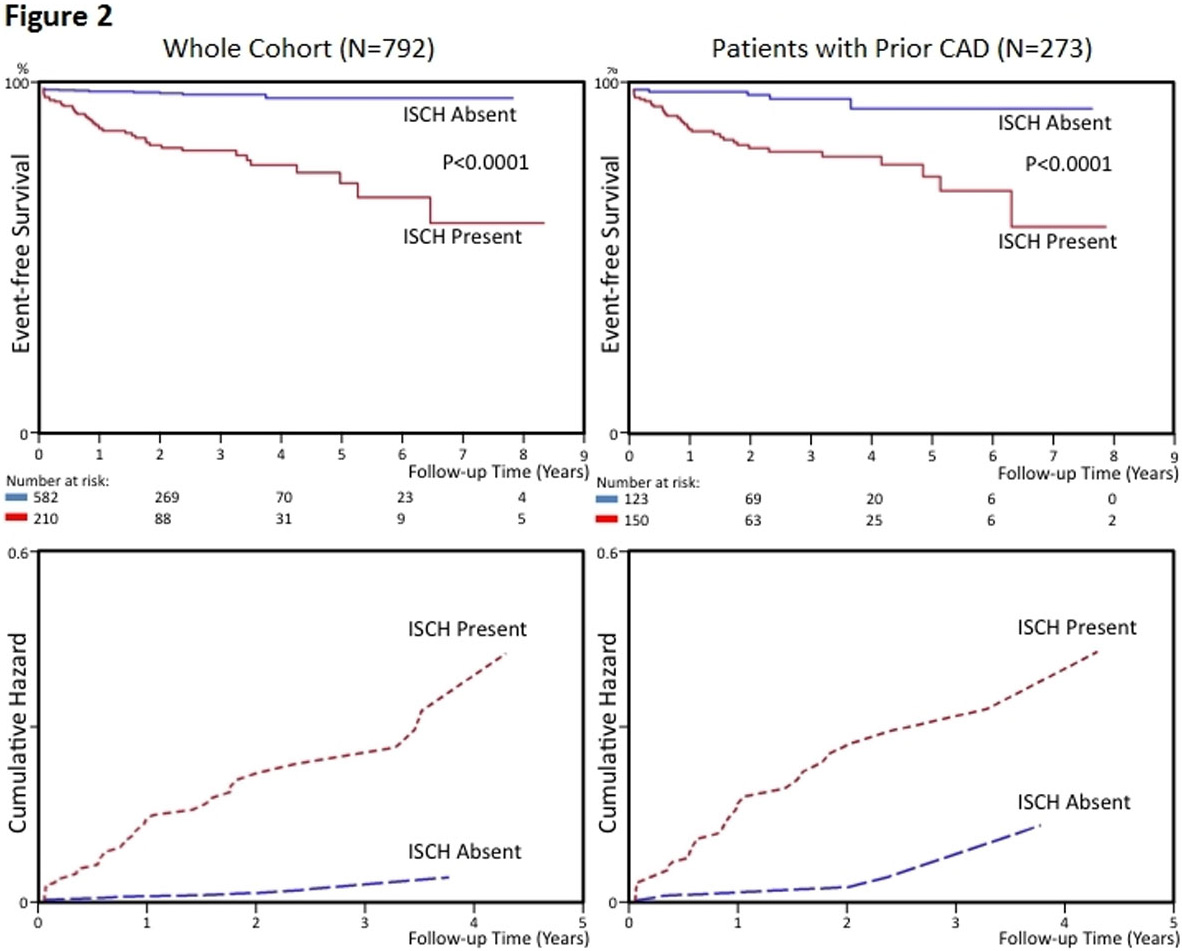
Kaplan-Meier MACE-free survival curves for the whole cohort (A) and patients with prior CAD (B). Follow-up period was truncated to 8 years. The bottom graphs (C and D) displayed the corresponding cumulative hazard function (bottom panels) for illustration of rates of MACE accumulation in these respective patient groups during the first 5 years after stress CMR assessment. P-value was derived using the log-rank test. Abbreviations: ISCH = inducible ischemia by stress CMR.

## Methods

In a prospective observational study of 815 consecutive patients clinically referred for stress CMR between 2001-2011, we studied the association of inducible ischemia on a composite of cardiac mortality and acute non-fatal myocardial infarction (MACE). We constructed Cox regression models to measure the independent and incremental association of inducible ischemia with our composite outcome beyond traditional markers of risk (including prior CAD). In addition, we quantified net reclassification improvement across guideline-based 3-year risk categories of MACE: low (<3%), moderate (3-9%), and high (>9%) by inducible ischemia by CMR, incremental to clinical risk models.

## Results

Inducible ischemia was the strongest predictor of MACE in the overall cohort and in patients with prior CAD (hazard ratios 15.66 and 8.18, respectively; P<0.0001). Absence of inducible ischemia was associated with low annual rates of MACE and cardiac death (whole cohort, 0.6% and 0.4%; prior CAD, 1.3% and 0.6%, respectively). Addition of inducible ischemia to the best clinical risk model (age and prior CAD adjusted) improved discrimination of MACE (C-statistic 0.81 to 0.86; P<0.0001). Inducible ischemia reclassified 91.5% of patients at moderate pre-test risk (65.7% to low risk; 25.8% to high risk), with corresponding changes in annual event rates (post-test low risk, 0.3%/year; post-test high risk, 4.9%/year). Categorical net reclassification index was 0.229 (95% 0.063-0.391). For patients with prior CAD, inducible ischemia reclassified 44% of patients at moderate pre-test risk and 37% at high pre-test risk to lower post-test risk, with a low annual rate of MACE (1.2%/year in both groups).

## Conclusions

The presence and extent of inducible ischemia by stress CMR effectively reclassifies patient risk beyond standard clinical risk factors, specifically in patients at moderate-to-high pre-test risk and in patients with prior CAD.

## Funding

Dr. Shah is supported by an American Heart Association Post-Doctoral Fellowship Award (11POST000002) and a training grant from the Heart Failure National Institutes of Health Clinical Research Network (U01-HL084877). Dr. Heydari is supported by a Clinical Fellowship Award from the Alberta Heritage Foundation for Medical Research. Dr. Murthy is supported by National Institutes of Health training grant T32-HL094301. Dr. Kwong is supported by a National Institutes of Health grant R01-HL091157 and a research grant from Astellas Pharmaceuticals.

